# Long COVID Optimal Health Programme to Enhance Mental and Physical Health: A Feasibility Randomised Controlled Trial

**DOI:** 10.1111/hex.70399

**Published:** 2025-08-21

**Authors:** Hiyam Al‐Jabr, David J. Castle, David R. Thompson, Karen Windle, John Belcher, Mónica M. De Icaza Valenzuela, Toby Helliwell, Chantal F. Ski

**Affiliations:** ^1^ School of Medicine Keele University Staffordshire UK; ^2^ Department of Research and Innovation Midlands Partnership University NHS Foundation Trust Stafford UK; ^3^ Department of Psychiatry University of Tasmania Tasmania Australia; ^4^ Centre for Mental Health Service Innovation Tasmania Australia; ^5^ School of Nursing and Midwifery Queen's University Belfast Belfast UK; ^6^ Centre for Applied Dementia Studies University of Bradford Bradford UK; ^7^ School of Medicine Keele University Keele UK; ^8^ Integrated Care Academy University of Suffolk Suffolk UK

**Keywords:** feasibility randomised controlled trial, long COVID, mental health, Optimal Health Programme

## Abstract

**Introduction:**

Long COVID (LC) is characterised by fatigue, muscle weakness and impaired concentration among other symptoms. No standardised diagnostic or treatment pathway is yet available, though a holistic, person‐centred approach to symptom management is recommended. The LC Optimal Health Programme (LC‐OHP) is a psychoeducational programme designed to support the mental and physical health of people with LC. This study aimed to examine the feasibility of delivering the LC‐OHP to people with LC.

**Methods:**

This was a feasibility randomised controlled trial of the LC‐OHP conducted across the UK. Adults diagnosed with LC were recruited and randomised to control (usual care) or to intervention (LC‐OHP) groups; follow‐up questionnaires were completed at three‐ and 6‐months (December 2021 to May 2023).

**Results:**

Sixty participants were recruited with a completion rate of 83% (*n* = 50). Most participants in the LC‐OHP group completed programme sessions (*n* = 19, 68%), rated the programme positively (*n* = 23, 87%); and felt that it had potential to improve health outcomes (*n* = 42, 70%). Initial findings demonstrate improvements across all variables at 3‐ and 6‐months, and more so for the LC‐OHP group than the control group in the short‐term.

**Conclusion:**

Findings support the feasibility of delivering the LC‐OHP to people with LC. Further, initial data demonstrate potential for the programme to improve most outcomes at three and 6 months. Data from this feasibility trial will be used as an evidence base to support a fully powered RCT of the LC‐OHP on patients with LC.

**Patient or Public Contribution:**

The LC‐OHP programme was adapted from the original OHP. Taking into account the various symptoms that people with LC experience, including fatigue and brain fog, public members were not directly involved in the design of the study; however, several approaches were considered to obtain ongoing support from public members while conducting the study, to suit people with LC. This included consulting with practitioners who care for people with LC and implementing their feedback, implementing prior feedback from patients with other chronic health conditions who used the OHP in previous studies, and collecting and implementing feedback from participants receiving this programme in this study. Additionally, two public members with lived LC experience were members of the data management committee that overviewed the study progress and provided continuous support. Public members and practitioners provided advice and guidance on different aspects related to the LC‐OHP programme and to the process of delivering it to study participants. This included making the programme concise, visual, colourful, and more user‐friendly, and adjusting and adapting the mode and timing of delivering the programme sessions (i.e., reduce the session duration, use convenient delivery methods) as preferred by study participants.

**Trial Registration:** ISRCTN trial register: registration number 38746119, https://doi.org/10.1186/ISRCTN38746119.

## 1 Introduction

Since the COVID‐19 pandemic, evidence has emerged that symptoms may persist for weeks/months beyond the acute infection, collectively referred to as ‘long COVID’ (LC) [1, 2, 3, 4]. According to the National Institute for Health and Care Excellence (NICE), LC encompasses both ongoing symptomatic COVID‐19 (4‐12 weeks) and post‐COVID‐19 syndrome ( ≥ 12 weeks) [5, 6]. It is estimated that within the first 2 years of COVID‐19, around 17 million people across Europe alone experienced LC, and millions are expected to live with it for years to come [7]. It is estimated in the United States that 7.5% of adults continue to have symptoms ≥ 3 months beyond the acute infection [8]. Further, evidence continues to demonstrate ongoing, persistent symptoms of LC globally [9, 10, 11, 12, 13]. In the United Kingdom, the number of people with symptomatic LC is estimated between one (persisting ≥ 4 weeks) [14] to two million (persisting ≥ 12 weeks) [15], with reports of one in ten [16, 17] and one in 20 people with persistent symptoms at ≥ 12 weeks [18].

Risk factors for LC are numerous, including gender, age, symptom severity in the acute phase, and concomitant chronic illnesses [19, 20, 21]. LC affects multiple organ systems with persistent and fluctuating symptoms with consequent restriction in quality of life (QoL) [22]. Most reported symptoms include fatigue, depression, anxiety, brain fog and muscle/joint pain [14, 23, 24, 25, 26, 27, 28]. The multitude of symptoms, global reach of the pandemic, and associated uncertainties have caused considerable distress and burden on healthcare systems, patients, and the public [29, 30, 31, 32].

To date, no evidence‐based standardised approach to managing LC exists. However, recommendations are to use comprehensive pathways for LC management [5, 33, 34]. Of note, with significant variation in LC presentation, it is recommended that different approaches should be considered to suit patients’ individual needs [34, 35, 36]. The initial priority in responding to the pandemic was to manage and prevent the acute infection. Considering the enormity of its impact (e.g., personal, social, economic, and health), the priority has shifted toward providing comprehensive support [23, 37]. Due to the increasing demands placed on mental health services by COVID‐19 and LC [23], along with workforce shortages and increasing multimorbidity, the sustainability of healthcare systems is increasingly under threat.

The Optimal Health Programme (OHP) is a psychoeducational self‐efficacy programme originally developed to support people with mental health issues [38, 39, 40]. This study aimed to examine the feasibility of delivering the LC‐OHP to people with LC. Primary objectives were to explore the feasibility (i.e., recruitment and retention rates) and acceptability (i.e., intervention adherence, completion of outcome assessments, acceptability and fidelity of the intervention, reasons for withdrawal) of delivering the LC‐OHP. Secondary objective was to identify any indicators of potential impact of the intervention on anxiety, depression, QoL, self‐efficacy and fatigue across groups and time through trends in data.

## 2 Materials and Methods

### 2.1 Ethics and Governance

Ethics approval was granted by the university research ethics committee (RETH21/004) and NHS Health Research Authority (IRAS no. 304234). The study was overseen by a Data Management Committee (DMC), which comprised 11 members (including public members, clinicians, and researchers), and met four times over the course of the study. Protocol amendments (e.g., expanding recruitment strategies) were approved by the relevant ethical committees.

### 2.2 Participants

Eligibility criteria were patients aged ≥ 18 years, confirmed diagnosis with COVID‐19 and LC (through reporting of PCR testing or confirmation by a clinician involved in the management of their LC); able to communicate in English and provide consent.

### 2.3 Participant Recruitment

**Figure 1:**
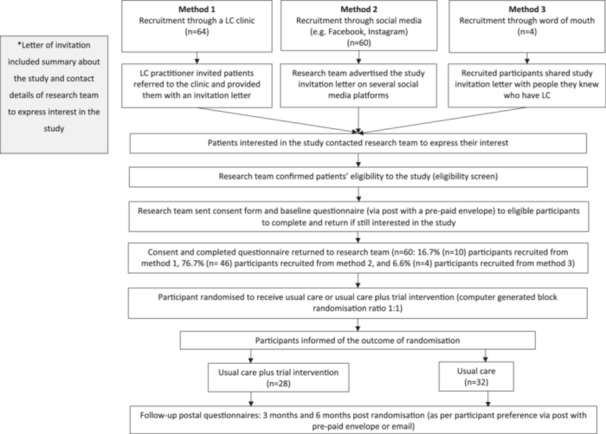
Flowchart of participant identification, invitation and recruitment.

Multiple recruitment approaches were used (Figure 1).

### 2.4 Participant Randomisation

Once consented, participants were randomised post‐baseline data collection by an independent researcher using computer‐generated block randomisation [43]. Due to the nature and length of the intervention, neither the researcher nor the participant was blinded to the treatment allocation.

### 2.5 Intervention

The OHP has been successfully implemented across a range of long‐term conditions [38, 39, 44, 45, 46]. The LC‐OHP addresses mental and physical dimensions of health and wellbeing [47]. It works on enhancing self‐efficacy and self‐management skills, with expectations of reductions in healthcare system burden and economic strain. In this study, the OHP was adapted to people with LC, LC‐OHP description is shown in Table 1.

**Table 1:** Description of LC‐OHP sessions.

Session	Title	Content
1	Optimal health	What is optimal health?
		*Optimal Health Wheel*
2	I‐Can‐Do‐Model	Strengths and Vulnerabilities
		Stressors and Strategies
		*Health Plans 1 & 2*
3	Factors of Wellbeing	Medication and metabolic monitoring
		Collaborative partners and strategies
		*Health Plan 3*
4	Visioning and Goal Setting	Defining change
		Orientation and preparation
		Creative problem solving and goal setting
		*Reflection & Celebration*
5	Building Health Plans	Health Plans 1, 2 and 3
		*My Health Journal*
Booster	Self‐reflection of learning on the journey to sustain wellbeing	Self‐reflection of learning on the journey to sustain wellbeing

### 2.6 Patient and Public Involvement

The OHP was refined using feedback provided by patients with long‐term conditions who used the programme in previous trials, to make it more succinct, colourful, and visual. Additionally, LC clinic practitioners (i.e., two occupational therapists) who were involved in recruiting participants also provided guidance regarding most convenient mode, time and duration of programme delivery as per patient needs.

Throughout the trial, the programme was further refined and adapted by two patient and public involvement (PPI) members of the DMC, with LC. Through monitoring and review of study progression, they gave feedback on programme session delivery (e.g., reducing session duration where needed to avoid fatigue), and advised and supported tailoring of content to suit the fluctuating nature of LC (e.g., paying attention to physical activity vs. energy levels). Also, at the beginning of the trial, the programme was tested with the first two recruited participants to identify any necessary adjustments before delivery to others. Potential of adaptations was discussed with the research team inclusive of the original OHP programme developer (D.J.C.), to gain consensus before implementation to ensure the programme was more user‐friendly i.e., conciseness (less wordy), visual appeal (more colourful).

### 2.7 Procedure

Participants were provided with a soft and a hard copy of the programme and sessions were delivered at their convenience regarding date, time, and delivery mode (telephone vs online). The LC‐OHP was delivered in one‐on‐one sessions over a minimum of five sessions (one session per week, up to 1 h per session), followed by a booster session delivered 3 months post‐final session. Most sessions were delivered by the lead researcher (H.A.), who received specialised LC‐OHP training over a 2‐day workshop by the programme developer (D.J.C.). Participants were advised they could withdraw from the study at any point. Those who withdrew were invited to an optional online short interview to identify reason(s) for withdrawal and offer suggestions for programme improvement.

### 2.8 Data Collection

Questionnaires in Table 2 were completed at baseline (pre‐randomisation), three‐, and 6‐months post‐randomisation, as per participant preference (i.e., either by post with a pre‐paid envelope or electronically by email).

**Table 2:** Questionnaires completed at baseline, 3 and 6 months.

Questionnaire	Outcome	Details
Patient Health Questionnaire (PHQ‐9)	Depression	Validated questionnaire composed of nine items with four response options; ‘not at all’ (scored as 0) to ‘nearly every day’ (scored as 3). Total scores range from 0 to 27, with scores of ≥ 5, ≥ 10, ≥ 15, representing mild, moderate and severe depression [48].
The Generalised Anxiety Disorder Assessment (GAD‐7)	Anxiety	A seven‐item self‐administered questionnaire with four‐point answer scale (0 to 3). Usually used as a screening tool and severity measure for generalised anxiety disorder (GAD) [49, 50].
General Self‐Efficacy Scale (GSE)	Self‐efficacy	A 10‐item, valid and reliable questionnaire with a four‐point Likert scale (‘not at all true’ to ‘exactly true’). It tests the individual's self‐efficacy. Higher scores are indicative of higher self‐efficacy [51].
EQ‐5D‐5L	Quality of life	A validated five‐item questionnaire with a 5‐item answers providing a generic measure on five health dimensions: mobility, self‐care, usual activities, pain/discomfort, and anxiety/depression [52]. The questionnaire also includes a visual analogue scale with scores ranging from 0 and 100 that reflect current health status [53].
Fatigue Assessment Scale (FAS)	Fatigue	A validated 10‐item questionnaire with a five point answer scale; never (scored as 1) to always (scored as 5) [54, 55].
Treatment Evaluation Inventory‐ short form (TEI‐SF)	Programme evaluation	An adapted version of the TEI‐SF [56] was completed by participants in the intervention group at 6‐months to assess the LC‐OHP. The adapted version composed of seven items, rated by a 5‐point Likert scale (strongly disagree to strongly agree).

### 2.9 Sample Size

Recommendations for sample size are mostly cited at 30–35 participants per group for pilot and feasibility studies [57, 58, 59, 60]. In this study, the target sample was to recruit a total of 60 participants over 9 months.

### 2.10 Statistical Analysis

Data for recruitment, adherence and retention rates, completeness of data and acceptability of the intervention are summarised using descriptive summary statistics.

Several feasibility measures exist [61], for the purpose of this study, the research team agreed that the trial feasibility to be assessed as per the following criteria:

1. Recruiting target sample (i.e., 60 participants within 9 months).
2. Acceptable retention rates; total dropout rate < 20% (to avoid risking the validity of the study [62, 63])
3. Intervention implementation and practicality, as reflected by the success in delivering intervention as intended, and by participants' adherence in attending the intervention sessions.
4. Intervention acceptability as reflected by participants' evaluations of the intervention their intervention acceptability scores.

These criteria would help in gathering suitable information to estimate key design features of a future definitive RCT on using the intervention programme in the LC population.

Means and standard deviations at 3 and 6 months are presented to identify any trends in continuous variables (anxiety, depression, QoL, self‐efficacy and fatigue). Change from baseline and Cohen's effect size for each follow‐up point is calculated using complete case data to assess sensitivity to change.

### 2.11 Design and Setting

A feasibility randomised controlled trial (RCT) of a programme tailored to support LC patients (LC‐OHP) was conducted at a UK university between December 2021 and May 2023. The study included collecting quantitative and qualitative data. This manuscript reports the quantitative data. The study protocol and qualitative findings are reported elsewhere [41, 42].

## 3 Results

A total of 128 patients were invited to participate (Figure 1), of whom 60 (47%) were recruited and randomised to the intervention group (intervention group) (*n* = 28) or control groups (control group) (*n* = 32).

### 3.1 Demographics

Of the 60 recruited participants, most had completed their undergraduate and/or postgraduate education (*n* = 42, 70%) and were of white ethnicity (*n* = 52, 87%), mostly white British (*n* = 44, 73%). Demographics were similar across groups (Table 3).

**Table 3:** Demographics of recruited participants (*n* = 60).

Demographic	Intervention (*n* = 28, 47%)	Control (*n* = 32, 53%)	Total (*n* = 60, 100%)
Age (mean (SD))	46 (13)	41 (12)	44 (13)
Gender			
Male	4 (7%)	6 (10%)	10 (17%)
Female	24 (40%)	25 (42%)	49 (82%)
other	NA	1 (2%)	1 (2%)
Ethnicity			
White British	23 (38%)	21 (35%)	44 (73%)
Other White background	4 (7%)	4 (7%)	8 (13%)
Asian or Asian British	1 (2%)	4 (7%)	5 (8%)
Black or Black British	0	1 (2%)	1 (2%)
Other mixed/multiple backgrounds	0	2 (3)	2 (3%)
Educational level			
Secondary education	1 (2%)	3 (5%)	4 (7%)
Postsecondary education	9 (15%)	5 (8%)	14 (23%)
Undergraduate and/or post‐graduate education	18 (30%)	24 (40%)	42 (70%)

### 3.2 Primary Outcomes

#### 3.2.1 Completion Rate and Withdrawals

The overall study completion rate was 83% (*n* = 50). Ten participants withdrew from the study: eight pre‐3‐month follow‐up (intervention group *n* = 5), and two from the control group did not return final questionnaires. The mean (SD) age of withdrawn participants was 42 (± 14), with the majority being females (*n* = 6, 60%) and of White ethnicity (*n* = 7, 70%).

#### 3.2.2 Interviews With Withdrawn Participants

Three withdrawn participants received between two and five sessions; two were interviewed (contact was lost with the third participant). Withdrawal was related primarily to LC health‐related issues. Other reasons included prioritising activities in consideration of energy levels, programme not offered as a cure, and some programme tasks perceived as an additional burden. Some described familiarity with most LC‐OHP topics, due to proactive self‐sourcing LC support materials whilst waiting for medical appointments. Timing of LC‐OHP delivery was also mentioned as a contributor to withdrawal, with consensus that the optimal time of delivery would be during early stages of LC.

Programme materials were described as well‐structured, easy to understand, and relevant. However, one participant felt it needed to be more flexible in the amount of time allocated to discuss the key concerns targeted by the programme. Also suggested, was to signpost people with LC within the programme to ‘reliable’ sources of information.

#### 3.2.3 Adherence to Programme Sessions

The LC‐OHP was delivered as intended, in a minimum of six sessions including the booster session, with sessions' durations adapted to suit individual needs of participants. Over half of the intervention group completed all programme sessions (*n* = 19, 68%). Three participants (11%) completed all except the booster session, and one (4%) completed four sessions. Three participants required seven sessions, and three other required eight, nine, and 10 sessions for completion. Despite expressing positive feedback on the programme, two participants withdrew before the first session (7%), two after completing the second session (7%), and one before the booster session (4%). Most participants received sessions remotely via videoconference using a convenient platform (e.g., Microsoft Teams, Skype, Zoom), and one received sessions via telephone.

#### 3.2.4 LC‐OHP Acceptability and Satisfaction

Acceptability of the LC‐OHP was evidenced by participants in the intervention group who completed the programme evaluation questionnaire TEI‐SF (*n* = 23, 82%) and by the low attrition rates across time (18% at 3 months; further 0% at 6 months). Of participants who completed the TEI‐SF, 87% reflected positive reactions towards the LC‐OHP i.e., its potential for improving wellbeing. For example, the LC‐OHP was perceived to be effective (87%) and that it has the potential to permanent improvements (70%).

#### 3.2.5 Secondary Outcomes

Initial data on effects of the LC‐OHP within groups at 3‐ and 6‐month follow‐up are presented in Supporting File S1. As this was a feasibility trial, the statistics presented are to identify trends in outcome data only. At both time points, the direction of scores for the intervention group was favourable across all outcomes from baseline to 3 and 6 months, with decreasing scores in depression (PHQ‐9), anxiety (GAD‐7) and fatigue (FAS), and increases shown in self‐efficacy (GSE) and QoL (EQ‐5D‐5L). Outcomes scores in the control group also demonstrated some improvements apart from self‐efficacy (GSE) at 6 months. Note, these data should be viewed cautiously as comparisons need to reflect baseline scores and retention rates.

## 4 Discussion

### 4.1 Summary

Various interventions have been designed to support LC, mostly focusing on managing specific symptoms [64, 65, 66]. However, to the best of our knowledge, this is the first study that introduces a comprehensive programme consistent with current recommendations for supporting LC.

This study aimed to explore the feasibility and acceptability of delivering the LC‐OHP to people with LC, and to identify variability in key outcome measures of anxiety, depression, QoL, self‐efficacy and fatigue across groups and time. The study was built on a hypothesis that the programme will be found feasible to be delivered to this population, to be acceptable, and to deliver improvements on the various outcomes. Study findings support the feasibility and acceptability of the programme. This is evidenced by several factors including recruiting the target number of participants, the low attrition rate, overall study completion and high adherence and retention rates, along with participant evaluations of the LC‐OHP, reflecting thus the implementation and practicality of the intervention. These initial data suggest the LC‐OHP has the potential to lead improvements across all outcomes at 3 and 6 months. Additionally, those in the intervention group seem to have more favourable outcomes in the short‐term (3 months) compared to the control group.

In this study, varied recruitment approaches were used including referral from LC clinics and social media to ensure recruitment of the target number of participants. Most participants were recruited using social media. The use of social media in recruiting research participants has been increasing and is expected to increase further [67]. Previous reviews reported the usefulness of social media platforms in recruiting participants for mental health research [68, 69]. Social media presents an effective and efficient platform to engage the community for research recruitment [70] and to facilitate researchers’ work [71]. Use of social media to recruit patients has been shown to have positive outcomes for scalability and reach, e.g. 38% of studies in a recent review included hard‐to‐reach participant population through the use of social media [71]. However, recruiting through social media could pose some challenges including limiting the age diversity of recruited participants, mostly with the elderly, who might not be highly engaged with these technological platforms. This has been reported by a recent review [71] and is also reflected in this study, as the average age of recruited participants was in their forties.

Additionally, of note, most recruited participants were of white ethnicity. There is inconsistent reporting with regard to ethnic groups most affected by LC. For example, some report this to be higher amongst the white non‐Hispanic population [72], whereas others report it to be higher among the blacks and Hispanic populations [73]. This may be due to challenges with diagnosing the condition, with many people living the condition without being diagnosed [74]. A recent systematic review reported that people of ethnic minority backgrounds are underrepresented or incorrectly classified in UK‐based LC trials [75]. The review also identified several barriers to recruiting this population, including language. This potentially contributed to recruiting a less diverse population in this trial, as we only included people who could communicate in English. Therefore, future research should implement further measures to overcome potential recruitment barriers (e.g., modifying language to suit diverse populations) and use wider recruitment approaches to increase reach and awareness about research and to encourage patients with varied demographic characteristics and ethnic backgrounds to participate, to identify the efficacy and acceptability of the programme on the various ethnic populations.

Similarly, most recruited participants were females. This is generally a common finding in research as females tend to participate in research more than males [76, 77, 78, 79]. This is also not surprising, since LC is reported to be more common among females [2, 19, 20, 29, 80, 81, 82, 83, 84, 85, 86, 87, 88]. Consideration should also be given to the stage of LC (i.e., duration of LC journey) when participants are recruited, and other supportive measures they are receiving. Time of contacting acute COVID‐19 infection and the duration of time of experiencing LC may have also impacted the findings, as people experience this illness differently and thus may have different reactions to the programme and other supportive measures they received. The duration of LC and other support offered/received by participants has not been collected as part of this study. Future research should therefore consider exploring this and identifying the impacts of delivering the programme at different stages of LC, in comparison to the different supportive measures offered to this population. Consideration should also be given to what constitutes usual care given to people with LC and the impact this may have on other supportive interventions.

With regard to the programme, the high adherence and satisfaction rates support acceptability of the LC‐OHP. Of note, withdrawn participants in the intervention group reported benefits from the programme and that it may have been of even greater benefit had it been introduced at an earlier stage. This is consistent with the views of other participants in the intervention group [42] who were interviewed at the end of the study [89], and with participants who received the original OHP, with similar suggestions to receive the programme early in the disease course [90].

The OHP was originally designed to be delivered in nine sessions and is highly flexible to the needs of individual patients [37, 39, 40, 47]. Our findings demonstrated that the LC‐OHP could be completed in at least six sessions dependent on participant's needs. This is akin to findings of a study where the OHP programme was delivered to people with diabetes [91]. The findings are also consistent with other studies that used the OHP in managing long‐term conditions, with positive findings reported by people who received it [39, 47, 90].

### 4.2 Strengths and Limitations

The LC‐OHP was adapted in consultation with an LC‐focused clinical team, following testing it with the first two participants, and was informed by interviews with a sample of the intervention group participants. Moreover, it was delivered according to participant preference and tailored to their needs. Most sessions were delivered by the same researcher, for the purpose of optimising fidelity. The sample represented a range of demographic characteristics, in terms of age, education, social, professional backgrounds and illness experience. Various recruitment approaches were used, which extended reach to participants across the United Kingdom. The study limitations included the following: over a third of participants in both groups were graduates/post‐graduates, perhaps restricting an assessment of how far the LC‐OHP could be successfully applied alongside those from deprived populations or who may have challenges with literacy. Most participants were recruited through social media, which might have limited the diversity of recruited participants, for example, regarding age (i.e., few elderly people were recruited) and ethnic backgrounds. The sample was predominantly white British; additionally, there was a higher proportion of female participants, yet this is consistent with LC literature [80, 92, 93]. With no standard treatment for LC, there may have been variability in supports received from other resources/health services. Additionally, public members with LC were not involved in refining the programme prior to starting the trial, which could have had derived further changed to the programme, thus causing a different experience. Also, for pragmatic purposes, as a preliminary study, the lead researcher also delivered the intervention. For a large‐scale, fully powered study, a clinical practitioner would also deliver the intervention for the purpose of scalability and external validity. Lastly, although the LC‐OHP may have contributed to the improvements in the intervention group, other factors may also have contributed, as some improvements were also reported by the control group. These include variations in usual care, other additional supports (e.g., from family and/or friends), and variations in severity and duration of LC.

## 5 Conclusion

A comprehensive, person‐centred approach is recommended to manage LC [5, 6]. The LC‐OHP was purposefully designed to be consistent with these recommendations. Findings demonstrate the feasibility and acceptability of the LC‐OHP in an LC population. Trends in the data show promise for improvements across a range of outcomes, thus supporting progression to conduct a fully powered RCT to identify the efficacy of the LC‐OHP on improving outcomes related to the mental and physical wellbeing across a more diverse LC population. Further investigation on the impacts of other supportive measures received by participants, particularly by those in the control group, and what constitutes ‘usual care’ is also recommended.

## Author Contributions

**Hiyam Al‐Jabr:** conceptualising, methodology, validation, formal analysis, investigation, resources, data curation, writing – original draft, writing – review and editing, visualisation, supervision, project administration. **David R. Thompson:** conceptualising, methodology, validation, resources, writing ‐ review and editing, supervision. **David J. Castle:** conceptualising, methodology, validation, resources, writing ‐ review and editing, supervision. **Chantal F. Ski:** conceptualising, methodology, validation, formal analysis, investigation, resources, data curation, writing ‐ review and editing, supervision, project administration, funding acquisition. **Karen Windle:** methodology, validation, formal analysis, investigation, resources, writing – review and editing, visualisation, supervision, project administration. **Monica M. De Icaza Valenzuela:** validation, formal analysis, investigation, writing – review and editing, project administration. **John Belcher:** validation, formal analysis, writing – review and editing. **Toby Helliwell:** validation, formal analysis, writing – review and editing.

## Ethics Statement

The study received ethical approval from the University of Suffolk research ethics committee and the Health Research Authority ethical committee in November 2021. All participants provided written informed consent before starting the study.

## Conflicts of Interest

The authors declare no conflicts of interest.

## Supporting information

Supporting file 1 Summary Data of Outcome Variables.

## Data Availability

The data that supports the findings of this study are available in the Supporting Information of this article. The data supporting the findings of this study are available from the corresponding author upon reasonable request.
