# Supplementary material for: Long COVID Optimal Health Programme to Enhance Mental and Physical Health: A Feasibility Randomised Controlled Trial
Source: Health Expect. 2025 Aug 21;28(4):e70399. doi: 10.1111/hex.70399 (PMC12368431; doi:10.1111/hex.70399)
Supplement: Supplementary file 1 — Supporting file 1 Summary Data of Outcome Variables. [file HEX-28-e70399-s001.docx]

**Supplementary file 1**

Summary Data of Outcome Variables

|  | **Intervention** | | | | **Control** | | | | |
| --- | --- | --- | --- | --- | --- | --- | --- | --- | --- |
| **Outcome Measure Time** | **N** | **Mean [SD]** | **Change [SD]** | **Effect**  **Size** | | **N** | **Mean [SD]** | **Change [SD]** | **Effect**  **Size** |
| PHQ-9 0 | 28 | 12.39 [4.79] |  |  | | 32 | 13.88 [6.71] |  |  |
| 3 | 23 | 10.01 [5.44] | -2.22 [2.98] | 0.74 | | 29 | 11.59 [6.25] | -2.07 [3.92] | 0.53 |
| 6 | 23 | 9.52 [6.69] | -2.69 [4.18] | 0.64 | | 27 | 9.70 [6.35] | -4.48 [6.22] | 0.72 |
| GAD-7 0 | 28 | 7.18 [5.59] |  |  | | 32 | 10.47 [5.48] |  |  |
| 3 | 23 | 5.87 [5.68] | -1.22 [3.37] | 0.36 | | 29 | 8.28 [5.71] | -2.31 [3.69] | 0.63 |
| 6 | 23 | 5.65 [6.34] | -1.43 [3.69] | 0.39 | | 27 | 7.19 [5.53] | -3.96 [5.84] | 0.68 |
| GSE 0 | 28 | 28.25 [5.49] |  |  | | 32 | 26.47 [6.46] |  |  |
| 3 | 23 | 28.87 [4.84] | 1.39 [3.85] | 0.36 | | 29 | 26.72 [6.33] | 0.66 [3.61] | 0.18 |
| 6 | 23 | 28.52 [7.20] | 1.04 [4.69] | 0.22 | | 27 | 25.01 [7.76] | -0.56 [4.32] | 0.13 |
| FAS 0 | 28 | 36.61 [7.14] |  |  | | 32 | 38.72 [7.37] |  |  |
| 3 | 23 | 34.35 [6.85] | -2.48 [4.46] | 0.56 | | 29 | 36.86 [7.89] | -2.21 [4.93] | 0.45 |
| 6 | 23 | 33.61 [7.14] | -3.22 [6.97] | 0.46 | | 27 | 34.74 [9.13] | -5.07 [8.34] | 0.61 |
| EQ-5D-DL: QoL 0 | 28 | 0.44 [0.25] |  |  | | 32 | 0.41 [0.26] |  |  |
| 3 | 23 | 0.56 [0.22] | 0.12 [0.15] | 0.80 | | 29 | 0.48 [0.25] | 0.09 [0.19] | 0.47 |
| 6 | 23 | 0.52 [0.29] | 0.08 [0.22] | 0.36 | | 27 | 0.48 [0.27] | 0.12 [0.22] | 0.54 |
| EQ-5D-DL: VAS 0 | 28 | 45.89 [20.18] |  |  | | 32 | 42.81 [19.09] |  |  |
| 3 | 23 | 50.95 [18.75] | 5.91 [14.42] | 0.41 | | 29 | 44.83 [19.98] | 2.93 [13.72] | 0.21 |
| 6 | 23 | 51.13 [22.06] | 6.09 [17.50] | 0.35 | | 27 | 50.59 [23.60] | 10.29 [17.11] | 0.60 |

EQ-5D-5L: EuroQol 5 Dimensions 5 Levels. FAS: Fatigue Assessment Scale. GAD7: Generalised Anxiety Disorder Assessment. GSE: General Self-Efficacy Scale. PHQ-9: Patient Health Questionnaire (PHQ-9) to measure depression. QoL: Quality of life. VAS: visual analogue scale.
